# Assessing the Frequency-Dependent Conductivity of Conductive Yarns

**DOI:** 10.3390/s26082554

**Published:** 2026-04-21

**Authors:** Balaji Dontha, Asimina Kiourti

**Affiliations:** ElectroScience Laboratory, Department of Electrical and Computer Engineering, The Ohio State University, Columbus, OH 43210, USA; balajidontha@gmail.com

**Keywords:** conductive threads, e-threads, flexible electronics, frequency-dependent conductivity, insertion loss, textile antennas

## Abstract

**Highlights:**

**What are the main findings?**

- This work establishes a validated, systematic framework for extracting both surface and bulk frequency-dependent conductivity of electrically conductive yarns over a broad Radio Frequency (RF) range (10 MHz–6 GHz) using transmission-line de-embedding methods.
- We identify frequency-specific performance limits and optimal usage regimes for different commercial conductive yarns, providing quantitative guidance for RF textile design.

**What are the implications of the main findings?**

- This work enables accurate electromagnetic modeling of conductive yarns and other low-conductivity materials, eliminating the need for unrealistic copper or Perfect Electric Conductor (PEC) assumptions in simulations.
- Our findings allow designers to predict different RF metrics, such as antenna efficiency, impedance matching, and radiation performance, more reliably, leading to improved first-pass design success and reduced prototyping cycles.

**Abstract:**

This study investigates the frequency-dependent electrical conductivity of electrically conductive threads (also known as e-threads), particularly focusing on their inherently lower conductivity than traditional conductors like copper. While efforts have been made to electrically characterize conductive threads in the past, most studies have focused on DC or frequencies lower than 1 GHz. Recent works have evaluated attenuation up to 6 GHz, but they do not report bulk conductivity and lack validation in the context of antenna applications. In a major step forward, this study reports a systematic way of characterizing the surface conductivity of conductive yarns, for eight different thread types, from 10 MHz to 6 GHz. Different parameters such as insertion loss, attenuation, and conductivity are reported, determining the suitability of conductive yarns at specific frequencies. The study also reports the first frequency-dependent bulk conductivity of individual conductive threads. By measuring both surface and bulk conductivity, our work provides foundational data crucial for designing textile-based antennas and sensors. The practical relevance of the proposed approach is demonstrated through simulations and measurements of a broadband log-spiral antenna and a single-turn loop antenna. Overall, this research contributes valuable insights into the integration of e-textiles in smart fabric applications, paving the way for further innovations in this evolving field.

## 1. Introduction

Rapid advances in wearable technology have driven a growing interest in the development of textile-based antennas and sensors in the Radio Frequency (RF) range. These devices, integrated into garments and other fabrics, often rely on electrically conductive threads, commonly referred to as conductive yarns or e-threads to transmit and receive signals. However, unlike traditional conductive materials (such as copper), the conductivity of these threads is inherently lower [1] and frequency-dependent, posing significant challenges in designing efficient RF components [2,3].

Indeed, as the operating frequency increases for functional conductive yarn surfaces, several loss mechanisms, such as skin effect and surface roughness, exacerbate the reduction in conductivity [4]. The skin effect, in particular, causes the current to be confined to a thin layer near the surface of the conductor at higher frequencies, effectively increasing the resistance and decreasing the overall conductivity. Surface roughness also contributes to increased resistance (particularly at higher frequencies, as the distance is comparable to the wavelength), further degrading performance [5]. These factors lead to a decline in the effectiveness of conductive yarns when used in RF antennas and sensors, resulting in altered impedance characteristics, reduced radiation efficiency, and suboptimal performance.

In [3], a dipole antenna developed for RF applications demonstrated significant discrepancies in performance between the copper- and conductive yarn-based fabrications. Such discrepancies in performance are particularly pronounced in multiband and broadband antennas [6]. In [7], patch antennas on flexible silver-coated threads and polydimethylsiloxane (PDMS) substrates exhibited a shift in operating frequency compared to their copper counterparts, along with a 2 dB reduction in realized gain. This effect was even more evident in antenna arrays [7]. Similarly, ref. [8] reported a shift in central frequency and an increase in bandwidth when using e-textile-based antennas compared to traditional metal-based microstrip patch antennas. In [9], a textile antenna for RF energy harvesting at 2.4 GHz demonstrated a gain reduction of over 1.5 dB and a 16% decrease in radiation efficiency. Similarly, in [10], a textile-based flexible rectenna operating in the 24 GHz millimeter-wave band showed significant deviations in reflection and transmission characteristics between the textile and copper prototypes. The transmission characteristics of textile Magneto-Inductive Waveguides (MIW) for on-body communications are also notably shifted in [11] as compared to the copper version. In [12], an ultrawideband (4 to 28 GHz) wearable textile antenna was reported for breast cancer detection, showing an average gain reduction of ~1 dB compared to simulation results that relied on copper. Likewise, ref. [13] discusses a textile antenna sensor for in vitro diagnostics of diabetes, demonstrating discrepancies between simulated and measured reflection coefficient magnitudes, along with a significant increase in bandwidth.

Although efforts have been made to electrically characterize conductive yarns at RF, results have been limited to DC [14] or frequencies lower than 1 GHz [15]. More recent works have extended these evaluations toward attenuation and surface conductivity studies up to 6 GHz [16], but they do not report bulk conductivity and lack validation in the context of antenna applications. Specifically, conductive yarns exhibit bulk conductivity, meaning that their conductive properties are distributed throughout the volume of the thread. When these conductive yarns are placed together to form a conductive surface (e.g., via embroidery approaches), it becomes more practical and relevant to define and evaluate their conductivity in terms of surface conductivity, i.e., the ability of a material to conduct electric current along its surface. For example, ref. [16] reports surface conductivity data from 100 MHz to 6 GHz, but does not report bulk conductivity data of the associated conductive yarns. The study also does not advance the validation of this data in the modeling, design, and simulation of antenna structures.

In a major step forward, we present a systematic framework to characterize the surface conductivity of conductive yarns from 10 MHz to 6 GHz and their bulk electrical conductivity from 900 MHz to 4 GHz, with representative frequencies used to compare attenuation, insertion loss, and validate performance in RF devices. Understanding the frequency-dependent electrical behavior of conductive yarns is crucial for the effective design and optimization of textile-based antennas and sensors: informed decisions can be made to improve performance, ultimately advancing the functionality and reliability of next-generation wearable technologies. Notably, knowledge of bulk conductivity of a single conductive yarn is equally important to knowledge of surface conductivity as several applications rely on single or standalone conductive yarns. A few examples include simple dipole and single-turn loop antennas; thread-based sensors for muscle atrophy [17]; single-turn electromagnetic (EM)-based loop sensors for acetone sensing [18]; and resonant loops for joint flexion monitoring [19]. Furthermore, the presented framework extends to other textile-integrated sensing platforms, such as wearable force-sensitive resistors [20] and conductive-thread-based electrodes for moisture sensing [21]. Beyond conductive yarns, the proposed approach can readily be expanded upon other conductive materials (fabrics or polymers) that exhibit lower conductivity than copper [22,23,24].

The rest of the paper is organized as follows. Section 2 details the employed microstrip transmission line (TL) technique and de-embedding methods employed to extract the frequency-dependent conductivity (both surface and bulk). Section 3 presents the results obtained from testing eight different conductive yarns. To validate our findings for surface and bulk conductivity, we employ a broadband spiral antenna and a single-turn loop antenna, respectively, in Section 4. The paper concludes in Section 5.

## 2. Materials and Methods

### 2.1. Embroidery and Conductive Threads

Embroidery, in the context of RF applications, refers to the technique of creating conductive patterns (e.g., antennas) on a substrate by automatically stitching conductive yarns into the fabric using an embroidery machine [25]. The versatility of embroidery is particularly advantageous when it comes to controlling the surface conductivity and impedance of the antenna. For example, by varying the conductive yarn type (material and thickness, among others) and by varying the number of threads per millimeter (i.e., line density), one can precisely manipulate the electrical characteristics of the embroidered structure. For instance, increasing the line density can enhance the surface conductivity, reducing the overall insertion loss and improving the antenna’s performance. Conversely, lower line densities may be used where more mechanical flexibility is required, allowing for more efficient material use, faster fabrication, and potentially lighter structures [7].

Among the several conductive yarns that are currently available in the market for embroidery, a few have proven to be particularly advantageous for RF applications and will be explored in this paper:

- Liberator 40/80 (Syscom, Columbus, OH, USA): This conductive yarn features a core made from Vectran, a liquid crystal polymer (LCP), which is renowned for its strength and thermal stability. For Liberator 40, the core comprises 40 closely packed filaments, each coated with conductive layers of silver, nickel, and copper with 80% of its composition by weight made up of these conductive metals. These coatings provide high electrical conductivity while maintaining a low DC resistance of 3.3 Ω/m, resulting in an overall yarn diameter of 0.023 cm. Similarly, the Liberator 80 conductive yarn, consisting of 80 filaments, has 78.5% of its composition by weight made up of the same conductive metals. The result is a yarn diameter of 0.033 cm and an even lower DC resistance of 1.97 Ω/m [26].
- Lyofil 66/99/166/332 (Syscom, Columbus, OH, USA): This conductive yarn features a core made from Zylon, a poly (p-phenylene-2, 6-benzobisoxazole) (PBO) fiber known for its exceptional strength, lightweight, and thermal stability. Each conductive yarn consists of X filaments (X stands for the number of filaments in Lyofil X), each coated with conductive layers of silver, copper, and nickel. Lyofil 66, 99, 166 and 332 conductive yarns have a yarn diameter of 0.013, 0.017, 0.025, and 0.036 cm, respectively, with 80% of their weight being composed of metal for Lyofil 66, 99 and 332, respectively, and 77% for Lyofil 166 [26].
- Agsis 100D (Syscom, Columbus, OH, USA): This conductive yarn is designed to have excellent flexibility, stretchability and washability and uses a nylon 66 fiber core with a coating of a conductive silver layer. It has a DC resistance of 65 Ω/m and a yarn diameter of 0.02 cm. The denier 100 D stands for the linear mass density of conductive yarns [26].
- Elektrisola (Elektrisola Feindraht AG, Escholzmat, Switzerland): This conductive thread is a fine metal wire that can be coated with enamel for insulation and protection. The core of the wire can be made from various metals such as copper, silver-plated copper, brass, or stainless steel, depending on the specific requirements for conductivity, flexibility, and durability. This paper uses silver-plated copper filaments bundled in 7 s to produce a single conductive thread that has a nominal diameter of 0.04 mm [27].

### 2.2. Fabrication of Transmission Lines

**Figure 1:**
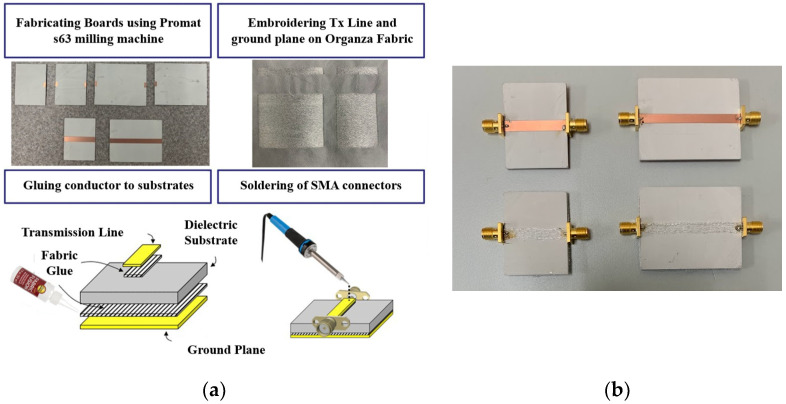
(**a**) Fabrication process of conductive yarn-based microstrip transmission lines; (**b**) 3 cm and 5 cm long fabricated copper and conductive yarn prototypes.

To assess the RF performance of conductive threads and extract conductivity parameters, TLs of varying lengths were employed, as detailed in Section 2.3 and Section 2.4. Specifically, 3 and 5 cm long, 4.5 mm wide microstrip TLs were fabricated on a Rogers 3003 substrate (Rogers Corporation, Chandler, AZ, USA), characterized by a dielectric constant (*ε_r_*) of 3.00 ± 0.04 and a loss tangent (*tanδ*) of 0.001 at 10 GHz with stable performance up to 77 GHz [28]. The fabrication process began with the Protomat S63 milling machine (LKPF Laser and Electronics, Garbsen, Germany), which was employed to precisely cut 4 cm wide rectangular pieces of the Rogers 3003 substrate. Subsequently, a Brother PR1X embroidery machine (Brother International Corporation, Bridgewater, NJ, USA) was used to embroider the TL structures on an organza fabric base (*ε_r_* of 1.03 and a *tanδ* of 0.005), utilizing a density of 4 conductive yarns/mm. This fabric base introduces a change of less than 1% to the effective substrate permittivity relative to Rogers 3003 alone. As such, the organza layer was not separately modeled in the de-embedding procedure, as its contribution to the extracted conductivity results is negligible. These embroidered TLs were then carefully adhered to the milled Rogers substrate. For comparison, copper TLs were also fabricated using 0.035 mm thick copper. To complete the microstrip TLs, SMA connectors were soldered at both ends. The overall fabrication process and resulting prototypes are illustrated in Figure 1.

### 2.3. Evaluating the Surface Conductivity of an Embroidered Surface

To determine the surface conductivity, we utilized the S-matrix de-embedding technique outlined in [16]. The technique employs two different lengths of TLs and copper reference to help eliminate the effects arising from the SMA connector junctions and dielectric losses due to the substrate. Return loss (***S***_11_) and insertion loss (***S***_21_) were measured using a PNA-L network analyzer (N5235A) (Keysight Technologies, Santa Rosa, CA, USA) over a frequency range of 10 MHz to 6 GHz. Initially, copper is selected. From the wave propagation theory of a lossy transmission line [4], we obtain

(1) $$S_{21,Li}^{eff}=\frac{V_{2}}{V_{1}}=A_{0}e^{-\gamma L_{i}},$$

where V_i_ represents the voltage at each port of the TL with length L_i_ and γ = α + jβ is the propagation constant. The $S_{21,L_{i}}$ includes the ***S***_21_ of the left/right pad junctions, while $S_{21,L_{i}}^{eff}$ is the effective S_21_ from the intrinsic line segment. To remove the pad junction effects, two different TLs of length l_1_ and l_2_ (l_2_ = l_1_ + ΔS) are used herein to obtain the intrinsic S_21_:

(2) $$S_{21,L_{2}-L_{1}(Cu)}=S_{21,L_{2}}\times S_{21,L_{1}}=e^{-\gamma ∆S},$$

As such, we can extract the propagation constant using:

(3) $$\gamma_{Cu}=\frac{\log\left(S_{21,L2-L1}\right)}{-∆S}=\alpha_{Cu}+j\beta_{Cu}$$

Here, the loss due to attenuation ($\alpha_{Cu}$) in a microstrip TL includes loss from the conductors ($\alpha_{Cond}$), loss in the dielectric substrate ($\alpha_{Dielectric}$), and loss from dispersion or radiation loss ($\alpha_{Rad}$):

(4) $$\alpha_{Cu}=\alpha_{Cond}+\alpha_{Dielectric}+\alpha_{Rad}$$

**Figure 2:**
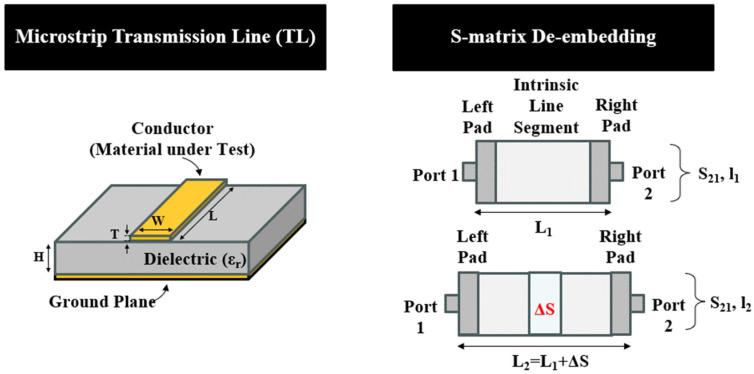
Microstrip transmission line (**left**) and S-matrix de-embedding process (**right**).

Since we know the conductivity of “gold standard” copper to be 5.8 × 10^7^ S/m, we can use the below Equations (5)–(9) to evaluate $\alpha_{Cond}$ for copper (namely, $\alpha_{Cond\_cu}$), where $R_{s}$ stands for surface resistivity, W and T are the dimensions of the microstrip TL, and H is the substrate height, as shown in Figure 2.

(5) $$G=0.94+0.132\frac{W}{H}-0.0032{\left(\frac{W}{H}\right)}^{2}$$

(6) $$R_{1}=\frac{R_{s}G}{W}\left(\frac{1}{\pi }+\frac{1}{\pi^{2}}\log\left(\frac{4\pi W}{T}\right)\right)$$

(7) $$R_{2}=\frac{R_{s}}{W}\left(\frac{W/H}{W/H+5.8+0.03H/W}\right)$$

(8) $$R_{s}=\sqrt{\frac{2\pi f\mu_{0}}{2\sigma }}$$

(9) $$\alpha_{Cond\_cu}=\frac{R_{1}+R_{2}}{2\times Z_{0}}$$

From here, we can determine losses other than the conductor ($\alpha_{Losses}$), as detailed in Equation (10).

(10) $$\begin{array}{cc} \alpha_{Losses} & =\alpha_{Dielectric}+\alpha_{Rad}\\ & =\alpha_{Total}-\alpha_{Cond\_Cu} \end{array}$$

This same procedure was applied to conductive yarn-based TLs. Since the values of $\alpha_{Dielectric}$ and $\alpha_{Losses}$ are fairly consistent in both copper and conductive yarn-based TLs, regardless of fabrication discrepancies, and we know $\alpha_{Cond\_Cu}$ from Equation (9), using Equations (1)–(11), we can extract the attenuation due to the conductive yarn alone ($\alpha_{Cond\_\mathrm{e-thread}}$) as follows.

(11) $$\alpha_{Cond\_\mathrm{e-thread}}=\alpha_{total\_\mathrm{e-thread}}-\alpha_{Losses}$$

Given that the conductive yarn exhibits significant surface roughness (denoted by *K*) compared to copper, the Hammerstad model [29] was applied. Equations (12) and (13) were utilized to evaluate the surface conductivity and effective attenuation, respectively, taking into account the increased surface roughness.

(12) $$K=1+\frac{2}{\pi }\tan^{-1}\left(1.4\times \frac{{∆}_{Surface\;Roughness}}{\delta_{Skin\;Depth}}\right)$$

(13) $$\alpha_{Cond-eff\_\mathrm{e-thread}}=\alpha_{Cond\_\mathrm{e-thread}}\cdot K$$

A surface roughness value of *K* = 2 is a good estimate for all conductive yarn types, consistent with values reported in the literature for rough conductive surfaces at microwave frequencies [5,16]. Using Equations (5)–(9), since all the quantities are known, we obtain R_s_e-thread_ and, finally, the frequency-dependent conductivity as:

(14) $$\sigma_{\mathrm{e-thread}}=\frac{2\pi f\mu_{0}}{2R_{s\_\mathrm{e-thread}}^{2}}$$

We have developed a MATLAB (version R2023a) (Mathworks. Inc, Natick, MA, USA) web application that implements the equations above to evaluate the surface conductivity of conductive yarns. The details regarding the application, including usage instructions and implementation, are provided in the Supplementary Information attached to this paper.

### 2.4. Evaluating the Bulk Conductivity of a Single Conductive Yarn

**Figure 3:**
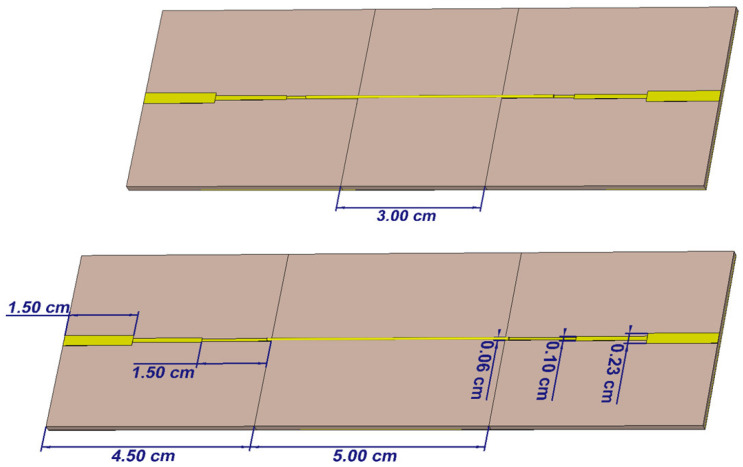
Three-section binomial matching network design in CST studio.

The surface conductivity model reported in Section 2.3 fails for single-yarn transmission lines that no longer exhibit a 50 Ω input impedance. To address this issue, a binomial multi-section matching network [4] was employed between the junction pads and TL. Figure 3 illustrates the use of a three-section matching network which assists in effectively evaluating the single conductive yarn conductivity from 900 MHz to 4 GHz. If desired, this range can be increased further by increasing the number of sections in the binomial matching network. However, this could lead to a decrease in accuracy of the obtained conductivity. The conductive yarn is positioned between the matching network sections. Next, the measurement and post-processing procedures outlined in Section 2.3 are employed to evaluate the bulk conductivity.

## 3. Results

### 3.1. Insertion Loss

Table 1 summarizes the insertion loss and attenuation due to conductor losses (α_cond-eff_e-thread_) for eight different conductive yarn types, with copper serving as the “gold standard” reference. The tests were conducted using a 4.5 mm wide and 5 cm long microstrip TL as a test case. As expected, copper exhibited the lowest insertion loss across all tested frequencies, ranging from 0.003 dB/cm at 100 MHz to 0.14 dB/cm at 6 GHz.

Liberator 40 and 80 demonstrated comparable insertion loss at 100 MHz, with values of 0.009 dB/cm and 0.008 dB/cm, respectively. However, at 6 GHz, the insertion loss for Liberator 80 increased significantly to 0.93 dB/cm, compared to 0.43 dB/cm for Liberator 40. This substantial increase for Liberator 80 can be attributed to its higher surface roughness and the skin effect, which becomes significant at higher frequencies. Although Liberator 80 contains more metal, its larger diameter results in increased surface roughness, leading to reduced effective surface conductivity. Similarly, the attenuation constant for Liberator 40 and 80 increased from 0.015 dB and 0.017 dB at 100 MHz to 1.089 dB and 2.511 dB at 6 GHz, respectively.

Within the Lyofil family, Lyofil 66 and 99 exhibited the lowest insertion loss at 100 MHz, both measuring less than 0.009 dB/cm. At 6 GHz, Lyofil 66 and 166 showed insertion losses of 0.66 dB/cm and 0.634 dB/cm, respectively, making Lyofil 66 the best performer in this category. Lyofil 166 and 332 performed better at intermediate frequencies, such as 915 MHz and 2.4 GHz, but their performance deteriorated significantly at higher frequencies. The attenuation for Lyofil 99 and 166 was the lowest at frequencies below 1 GHz, but at higher frequencies, Lyofil 66 outperformed the other members of the Lyofil family. It is important to note that the thickness of these conductive yarns increases from Lyofil 66 to 332, leading to a more pronounced skin effect and increased surface roughness.

Agsis 100D exhibits a relatively high insertion loss of 0.017 dB/cm at 100 MHz, primarily attributed to its elevated DC resistance of 65 Ω/m [26]. Notably, the insertion loss and attenuation show only a gradual increase with frequency, reaching 0.69 dB/cm and an attenuation of 1.76 dB/cm at 6 GHz. This behavior may be influenced by the yarn’s smaller diameter, which could reduce losses related to surface roughness at higher frequencies.

Elektrisola outperformed all other conductive yarns at lower frequencies, such as 100 MHz and 915 MHz, with an insertion loss of only 0.006 dB/cm and an attenuation of 0.001 dB at 100 MHz. This is because Elektrisola is mostly a thin enamel-coated metal and its diameter is very small compared to the other conductive yarns. It is important to note the insertion loss at 100 MHz is lower than copper. This anomaly at very low frequencies arises from limitations in the de-embedding procedure. The differential method uses transmission lines of 3 cm and 5 cm (Δ*S* = 2 cm), for which the insertion loss at 100 MHz is extremely small and approaches the measurement sensitivity limit. Consequently, the extracted attenuation for Elektrisola conductive yarn appears artificially low (even below copper), which is not physical but a result of measurement limitations. However, at 6 GHz, Liberator 40 emerged as the best performer in terms of both insertion loss and attenuation constant. This superior performance is likely due to its lower surface roughness, which reduces resistive losses caused by the skin effect at higher frequencies.

### 3.2. Frequency-Dependent Surface Conductivity

**Figure 4:**
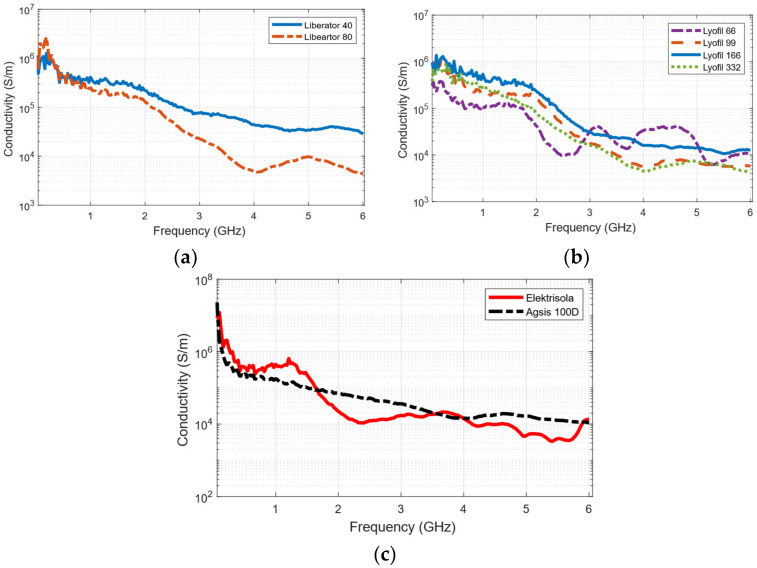
Frequency-dependent conductivity of: (**a**) Liberator 40 and 80, (**b**) Lyofil 66, 99, 166 and 332, and (**c)** Elektrisola and Agsis 100D.

Figure 4 provides a comparative analysis of the conductive yarns under investigation. In the Liberator family, Liberator 80 exhibited higher conductivity than Liberator 40 at frequencies below 1 GHz, which can be attributed to its greater metal content. However, at frequencies above 1 GHz, the larger diameter of Liberator 80 leads to increased skin effect and surface roughness, causing a significant signal attenuation and, thus, reduction in conductivity.

Similarly, within the Lyofil family, Lyofil 166 and Lyofil 332 demonstrated high conductivity, approximately 10^6^ S/m, at frequencies below 1 GHz. Beyond this range, their conductivity gradually decreases, with a pronounced drop beyond 2.4 GHz, eventually falling below 10^4^ S/m. Among the Lyofil conductive yarns, Lyofil 166 strikes a favorable balance between thread thickness and metal content, resulting in superior conductivity.

The Elektrisola threads demonstrated the highest conductivity among the tested conductive yarns, ranging from 10^6^ S/m to 10^7^ S/m at frequencies below 1 GHz. In contrast, Agsis threads exhibited conductivity below 10^6^ S/m in the same frequency range. Similar to other conductive yarns, the conductivity of both Elektrisola and Agsis decrease as the frequency increases.

### 3.3. Effect of Stitch Density on Surface Conductivity

To understand the effect of stitch density on surface conductivity, Elektrisola was considered, and three different stitch densities were compared, i.e., 1, 4 and 7 conductive yarns/mm were used for embroidering the ground plane and TL.

**Figure 5:**
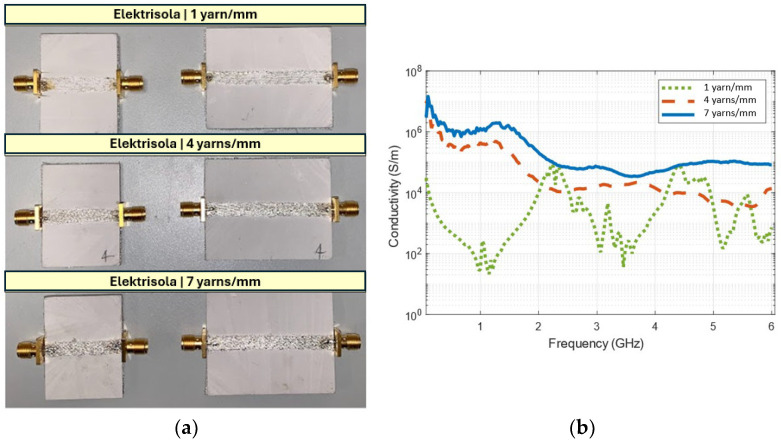
(**a**) Fabricated Elektrisola microstrip TL of various thread densities, and (**b**) frequency dependent conductivity.

Figure 5a shows the fabricated microstrip TL and Figure 5b shows the evaluated surface conductivity. With a stitch density of 1 yarn/mm, it becomes difficult to evaluate conductivity as the TL structure is not densely embroidered, leading to non-uniform current distribution and discontinuities in galvanic contact between adjacent stitches. The surface conductivity of 4 yarns/mm was ~10^6^ S/m at lower frequencies, but dropped to below 10^4^ S/m beyond 4 GHz. In contrast, the surface conductivity of 7 yarns/mm was higher even at high frequencies as the diameter of Elektrisola conductive yarns is only 0.04 mm. The finer diameter of Elektrisola conductive yarns contributed to a smoother surface, minimizing the impact of surface roughness and smoother current paths. As a result, the increased metal content with densely packed embroidery leads to less signal attenuation and, thus, delivers superior surface conductivity.

### 3.4. Single Conductive Yarn Bulk Conductivity

**Figure 6:**
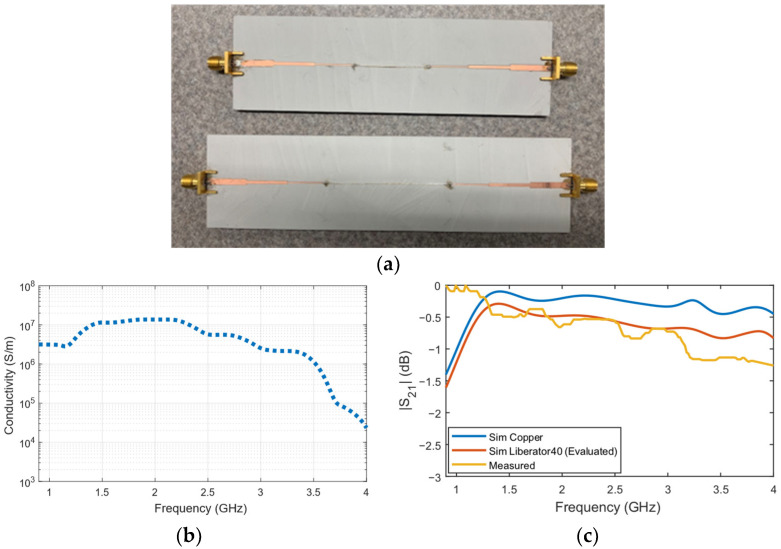
(**a**) Fabricated single e-thread microstrip transmission lines, (**b**) frequency-dependent conductivity, and (**c**) comparison of simulated and measured |S_21_| values.

To evaluate the conductivity of a single conductive yarn, Liberator 40 was selected as an example case. Figure 6a illustrates the fabricated single conductive yarn TLs with lengths of 3 cm and 5 cm connected between the matching networks. Figure 6b presents the evaluated conductivity, which remains stable up to 3.5 GHz, beyond which a significant drop is observed.

For the 5 cm TL, the insertion loss of the Liberator 40 was compared with that of a copper wire of the same diameter. At 915 MHz, the insertion loss was 0.009 dB/cm and 0.018 dB/cm for copper and Liberator 40, respectively. At 2.4 GHz and 4 GHz, the insertion loss for the copper wire increased to 0.138 dB/cm and 0.142 dB/cm, respectively, while for the Liberator 40, the values were 0.247 dB/cm and 0.322 dB/cm, respectively. The obtained conductivity was then used as an input to model the single-wire transmission line in CST microwave studio (Dassault Systèmes’, Waltham, MA USA), and the simulated |S_21_| closely matched the measured values up to 3 GHz, as shown in Figure 6c. These results confirm the validity of the approach.

## 4. Validation Using Antennas

### 4.1. Broadband Log-Spiral Antenna

**Figure 7:**
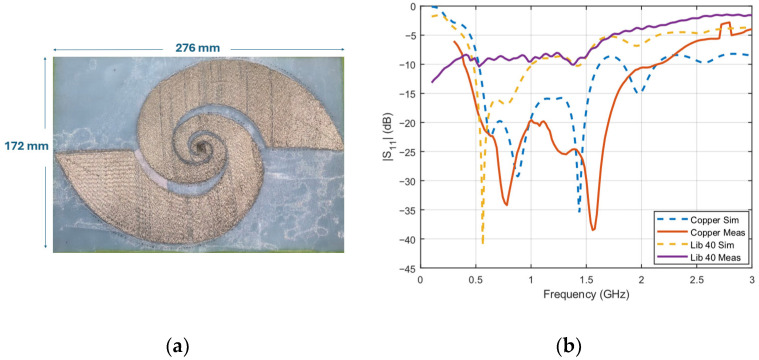
(**a**) Embroidered log-spiral antenna, and (**b**) reflection characteristics.

To thoroughly investigate the obtained surface conductivity of conductive yarns over a wide range of frequencies, a log-spiral antenna operating in the 400 MHz to 3 GHz range was selected. Figure 7a illustrates the antenna fabricated using Liberator 40 conductive yarn embroidered on an organza substrate. The logarithmic spiral antenna is defined by $r(\theta )=r_{0}e^{k\theta }$, where $r_{0}=9.54\;\mathrm{mm}$ and k=0.101. The spiral extends to a maximum radius of approximately 139 mm, corresponding to an overall diameter of 276 mm, and comprises approximately 4.4 turns with a feed gap of 1.2 mm. The antenna is backed by a PDMS substrate, for which frequency-dependent dielectric properties (*ε_r_* = 2.72 and *tan δ* = 0.05) were employed in CST Microwave Studio (MWS). The frequency-dependent conductivity, as determined in Section 3.2, was imported into CST MWS, with simulations conducted using the finite element method in the frequency domain solver.

As shown in Figure 7b, the simulated and measured results for the copper-based antenna demonstrate close agreement, validating the accuracy of the simulation model. Three independent measurements were performed, and the maximum deviation was 1.23 dB observed around 330 MHz. For the textile-based antenna using Liberator 40, the simulation results closely match the measurements, with the exception of frequencies below 500 MHz. This deviation can be attributed to the limitations in fabrication precision and the challenges of achieving the desired design accuracy with the selected conductive yarn type. Additionally, the measured reflection coefficient (|*S*_11_|) response does not exhibit the sharp dip around 600 MHz observed in the simulation. This discrepancy is likely due to a combination of fabrication tolerances, lower Q-factor of conductor, and inherent system losses. The primary objective of the antenna validation is to demonstrate that using experimentally extracted conductivity values produces simulation results closer to measurement than conventional copper or PEC assumptions, which is confirmed in Figure 7b. The remaining deviation below 500 MHz is attributed to the lower Q-factor of Liberator 40 (a direct consequence of its lower conductivity characterized in Section 3.2) which broadens resonant features and suppresses the sharp |*S*_11_| dip near 600 MHz observed in the simulation.

Notably, the simulation using the extracted Liberator 40 conductivity agrees more closely with the measured results than either the copper or PEC simulation, confirming the importance of using the experimentally extracted conductivity values for accurate prediction of conductive yarn-based antenna performance. The antenna validation presented here serves as a proof-of-concept demonstrating the agreement between simulation and measurement using the extracted conductivity values, and detailed performance metric comparisons including gain and radiation efficiency measurements are identified as future work.

### 4.2. Single-Turn Loop Antenna

**Figure 8:**
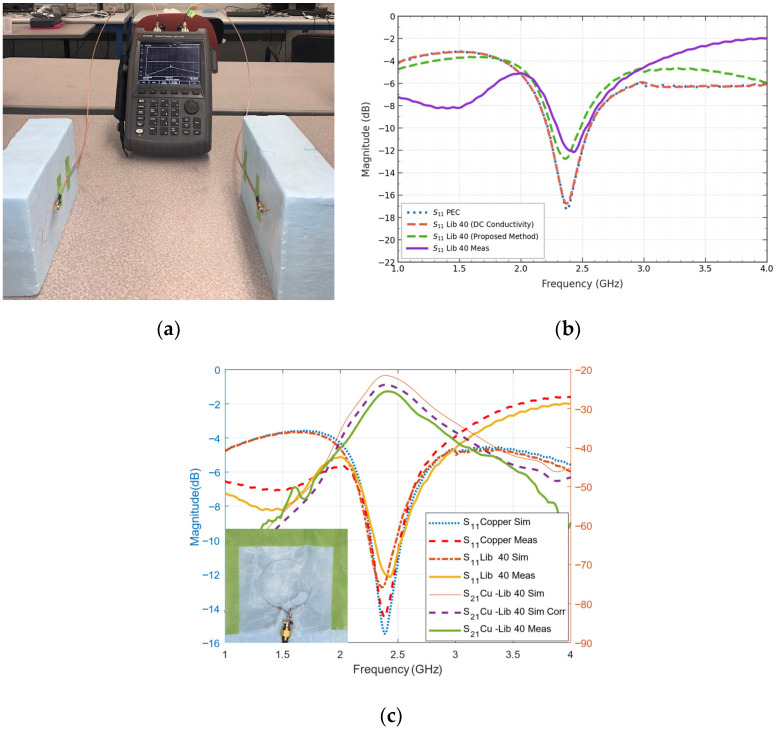
(**a**) Measurement setup of loop antennas (copper on left and Liberator 40 on right), (**b**) reflection characteristics using proposed method and (**c**) reflection and transmission characteristics.

To validate our findings, we selected a single-turn loop antenna of diameter 42 mm with a feed gap of 2 mm operating at 2.4 GHz fabricated using Liberator 40 conductive yarn on organza fabric. The obtained frequency-dependent bulk conductivity of Liberator 40 derived in Section 3.4 was imported in CST Microwave Studio as a new material and the finite element method was used. Figure 8a shows the measurement setup. Three antenna measurements were conducted for repeatability and the maximum change in both |*S*_11_| and |*S*_21_| combined was 1.01 dB at 2.52 GHz. Since this deviation is very small compared to the dynamic range, error bars were not included. Figure 8b highlights the difference in |*S*_11_| using PEC or DC conductivity of Liberator 40 for simulation (i.e., manufacturer specifications) compared to the proposed method. From Figure 8c, it can be observed that the simulation results based on the imported conductivity match closely with the measurement, confirming the accuracy of the obtained conductivity value. This proves that using the obtained values in simulation could help improve the design and optimization of conductive yarn or textile-based antenna designs.

## 5. Conclusions

This research provided a comprehensive analysis of the insertion loss, attenuation, and frequency-dependent surface conductivity of various conductive yarns across 100 MHz to 6 GHz. Our approach also provided a method to evaluate the bulk conductivity of a single conductive yarn using an accompanying binomial matching network.

The results demonstrated that some conductive yarns, such as Liberator 40 and Elektrisola (4 yarns/mm), exhibit promising performance at lower frequencies. However, their conductivity significantly diminishes as the frequency increases. This decline is primarily attributed to the skin effect and increased surface roughness at higher frequencies. Certain conductive yarns, like Lyofil 166 and 332, exhibit better performance at intermediate frequencies, but experience a notable drop in conductivity beyond 2.4 GHz. It was concluded that Liberator 40 is well-suited for applications operating below 1 GHz. However, for broadband applications extending up to 4 GHz and above, Elektrisola with an increased line density (7 yarns/mm) is preferable, as the denser embroidery effectively minimizes attenuation at higher frequencies.

Validation using a broadband log-spiral antenna and a single-turn loop antenna confirmed the practical applicability of our conductivity measurements. The close agreement between effective conductivity simulation and measurement results highlighted the reliability of the extracted conductivity values and emphasizes the potential of these findings to enhance the design of conductive yarn-based antennas accounting for such lower conductivity.

While this study characterizes eight representative conductive yarn types, the proposed framework is broadly applicable and can be readily extended to other widely used conductive yarns, such as silver-coated polyamide multifilament yarns (e.g., Shieldex 117/17 2-ply HCB), providing a versatile tool for the RF-textile community. It is further noted that combining the proposed electrical characterization framework with mechanical testing including tensile strength, bending, and wash cycle assessments, as performed in [30], would provide a comprehensive evaluation of conductive yarn’s suitability for real-world wearable applications. In conclusion, this research offers valuable insights into the frequency-dependent behavior of conductive yarns, paving the way for more effective and informed integration of e-textiles in advanced electromagnetic applications.

## Figures and Tables

**Table 1:** Insertion loss and attenuation constant in conductive yarns at different frequencies of interest.

Parameter	Conductive Yarn Type	Frequency					
		100 MHz	403 MHz	915 MHz	2.4 GHz	4 GHz	6 GHz
Insertion Loss (dB/cm)	Copper	0.0037	0.0132	0.0194	0.0393	0.1025	0.1477
	Liberator 40	0.0095	0.0274	0.0481	0.1263	0.3150	0.4388
	Liberator 80	0.0084	0.0241	0.0544	0.1732	0.7572	0.935
	Lyofil 66	0.0088	0.0397	0.0743	0.3648	0.3268	0.6606
	Lyofil 99	0.0064	0.0246	0.0544	0.1803	0.708	0.874
	Lyofil 166	0.0067	0.0226	0.0437	0.1446	0.4582	0.6347
	Lyofil 332	0.0095	0.0262	0.0454	0.2151	0.7795	0.9506
	Elektrisola	0.0062	0.0285	0.0461	0.3699	0.4812	0.8457
	Agsis100D	0.0177	0.0522	0.0818	0.2062	0.5032	0.69
Attenuation Constant (dB/cm)	Copper	0.0062	0.0196	0.0296	0.0483	0.0624	0.0751
	Liberator 40	0.017	0.0537	0.1096	0.3149	0.8401	1.0891
	Liberator 80	0.0158	0.0451	0.1288	0.4508	2.1138	2.5179
	Lyofil 66	0.0250	0.0983	0.1944	1.0104	0.8834	1.7365
	Lyofil 99	0.0143	0.0507	0.1332	0.4753	1.9812	2.4043
	Lyofil 166	0.0109	0.0432	0.1006	0.3711	1.2557	1.6562
	Lyofil 332	0.0241	0.0562	0.108	0.5764	2.1832	2.568
	Elektrisola	0.0014	0.0495	0.0966	1.0087	1.3112	2.2526
	Agsis100D	0.0011	0.0789	0.1606	0.4988	1.336	1.7657

## Data Availability

The original contributions presented in this study are included in the article/Supplementary Material. Further inquiries can be directed to the Asimina Kiourti or Balaji Dontha.

## Supplementary Materials

The following supporting information can be downloaded at https://www.mdpi.com/article/10.3390/s26082554/s1: MLAPP File: Conductivity_ethreads_APP; Figure S1: Matlab_App_Design_Review; Input Files: LYOFIL_66_3CM.s2p, LYOFIL_66_5CM.s2p, COPPER_3CM.s2p, COPPER_5CM.s2p.

## References

[1] Raji R.K., Miao X., Boakye A. (2017). Electrical conductivity in textile fibers and yarns—Review. AATCC J. Res..

[2] Mahfuz M.M.H., Islam M.R., Park C.W., Elsheikh E.A., Suliman F.M., Habaebi M.H., Malek N.A., Sakib N. (2022). Wearable textile patch antenna: Challenges and future directions. IEEE Access.

[3] Wang Z., Zhang L., Volakis J.L. (2013). Textile antennas for wearable radio frequency applications. Text. Light Ind. Sci. Technol..

[4] Pozar D.M. (2004). Microwave Engineering.

[5] Morgan S.P. (1949). Effect of surface roughness on eddy current losses at microwave frequencies. J. Appl. Phys..

[6] Wang Z., Lee L.Z., Psychoudakis D., Volakis J.L. (2014). Embroidered multiband body-worn antenna for GSM/PCS/WLAN communications. IEEE Trans. Antennas Propag..

[7] Wang Z., Zhang L., Bayram Y., Volakis J.L. (2012). Embroidered conductive fibers on polymer composite for conformal antennas. IEEE Trans. Antennas Propag..

[8] Liu N., Lu Y., Qiu S., Li P. (2011). Electromagnetic properties of electro-textiles for wearable antenna applications. Front. Electr. Electron. Eng. China.

[9] Zada M., Iman U.R., Basir A., Yoo H. (2024). Battery-free digitally embroidered smart textile energy harvester for wearable healthcare IoTs. IEEE Trans. Ind. Electron..

[10] Wagih M., Hilton G.S., Weddell A.S., Beeby S. (2020). Broadband millimeter-wave textile-based flexible rectenna for wearable energy harvesting. IEEE Trans. Microw. Theory Tech..

[11] Jenkins C.B., Kiourti A. (2023). Wearable dual-layer planar magnetoinductive waveguide for wireless body area networks. IEEE Trans. Antennas Propag..

[12] Mahmood S.N., Ishak A.J., Saeidi T., Soh A.C., Jalal A., Imran M.A., Abbasi Q.H. (2021). Full ground ultra-wideband wearable textile antenna for breast cancer and wireless body area network applications. Micromachines.

[13] Gharbi M.E., Fernández-García R., Gil I. (2021). Textile antenna-sensor for in vitro diagnostics of diabetes. Electronics.

[14] Stavrakis A.K., Simić M., Stojanović G.M. (2021). Electrical Characterization of Conductive Threads for Textile Electronics. Electronics.

[15] Shawl R.K., Long B.R., Werner D.H., Gavrin A. (2007). Characterization of conductive textile materials intended for radio frequency applications. IEEE Antennas Propag. Mag..

[16] Wang Z. (2014). Electronic Textile Antennas and Radio Frequency Circuits for Body-Worn Applications. Ph.D. Thesis.

[17] Rice A., Kiourti A. (2023). A stretchable, conductive thread-based sensor for wearable monitoring of muscle atrophy. IEEE Trans. Biomed. Eng..

[18] Dontha B., Faltas M., Gouma P.-I., Kiourti A. (2022). Electromagnetic-based deformation monitoring for PANI-CA breath acetone sensors. IEEE J. Electromagn. RF Microw. Med. Biol..

[19] Mishra V., Kiourti A. (2019). Wrap-around wearable coils for seamless monitoring of joint flexion. IEEE Trans. Biomed. Eng..

[20] Dontha B., Swearingen K., Swearingen S., Thrane S.E., Kiourti A. (2022). Wearable Sensors Based on Force-Sensitive Resistors for Touch-Based Collaborative Digital Gaming. Sensors.

[21] Qureshi S., Stojanović G.M., Simić M., Jeoti V., Lashari N., Sher F. (2021). Silver Conductive Threads-Based Embroidered Electrodes on Textiles as Moisture Sensors for Fluid Detection in Biomedical Applications. Materials.

[22] Dontha B., Moulod M., Balbaugh S., Hoelzle D., Li J., Miranda F.A., Kiourti A. (2024). RF characterization of a photocurable PEDOT:PSS:PEGDA conductive biomaterial for 3D-printing implantable antennas. IEEE Trans. Antennas Propag..

[23] Curry F., Chrysler A.M., Tasnim T., Shea J.E., Agarwal J., Furse C.M., Zhang H. (2020). Biostable conductive nanocomposite for implantable subdermal antenna. APL Mater..

[24] Adib A.A., Sheikhi A., Shahhosseini M., Simeunović A., Wu S., Castro C.E., Zhao R., Khademhosseini A., Hoelzle D.J. (2020). Direct-write 3D printing and characterization of a GelMA-based biomaterial for intracorporeal tissue engineering. Biofabrication.

[25] Alharbi S., Chaudhari S., Inshaar A., Shah H., Zou C., Harne R.L., Kiourti A. (2018). E-textile origami dipole antennas with graded embroidery for adaptive RF performance. IEEE Antennas Wirel. Propag. Lett..

[26] Syscom Advanced Materials. Metal-Clad Fibers. http://www.metalcladfibers.com.

[27] Elektrisola Feindraht AG Textile Wire GmbH. https://www.textile-wire.ch/en/home.html.

[28] Rogers Corporation (2024). RO3000^®^ Series Circuit Materials Datasheet.

[29] Hammerstad E., Jensen O. (1980). Accurate models for microstrip computer-aided design. Proceedings of the IEEE MTT-S International Microwave Symposium.

[30] Mersch J., Winger H., Altinsoy E., Cherif C. (2023). Electromechanical Properties of Silver-Plated Yarns and Their Relation to Yarn Construction Parameters. Polymers.

