# Supplementary figures and images for: Assessing the Frequency-Dependent Conductivity of Conductive Yarns
Source: Sensors (Basel). 2026 Apr 21;26(8):2554. doi: 10.3390/s26082554 (PMC13120523; doi:10.3390/s26082554)

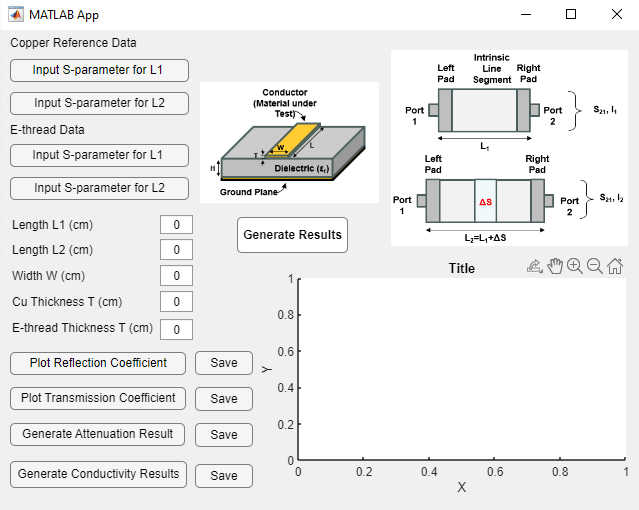

Supplement: Supplementary file 1 [file sensors-26-02554-s001.zip › Matlab_App_Design_Preview.png]
